# Better Estimation of Spontaneous Preterm Birth Prediction Performance through Improved Gestational Age Dating

**DOI:** 10.3390/jcm11102885

**Published:** 2022-05-19

**Authors:** Julja Burchard, George R. Saade, Kim A. Boggess, Glenn R. Markenson, Jay D. Iams, Dean V. Coonrod, Leonardo M. Pereira, Matthew K. Hoffman, Ashoka D. Polpitiya, Ryan Treacy, Angela C. Fox, Todd L. Randolph, Tracey C. Fleischer, Max T. Dufford, Thomas J. Garite, Gregory C. Critchfield, J. Jay Boniface, Paul E. Kearney

**Affiliations:** 1 Sera Prognostics, Incorporated, Salt Lake City, UT 84109, USA; ashoka@seraprognostics.com (A.D.P.); rtreacy@seraprognostics.com (R.T.); afox@seraprognostics.com (A.C.F.); trandolph@seraprognostics.com (T.L.R.); tfleischer@seraprognostics.com (T.C.F.); mdufford@seraprognostics.com (M.T.D.); tgarite@seraprognostics.com (T.J.G.); gcritchfield@seraprognostics.com (G.C.C.); jboniface@seraprognostics.com (J.J.B.); pkearney@seraprognostics.com (P.E.K.); 2 Department of Obstetrics & Gynecology, The University of Texas Medical Branch, Galveston, TX 77555, USA; gsaade@utmb.edu; 3 Department of Obstetrics and Gynecology, Division of Maternal-Fetal Medicine, University of North Carolina, Chapel Hill, NC 27599, USA; kim_boggess@med.unc.edu; 4 Maternal Fetal Medicine, Boston University School of Medicine, Boston, MA 02118, USA; glenn.markenson@bmc.org; 5 Department of Obstetrics & Gynecology, The Ohio State University, Columbus, OH 43210, USA; jdiamsmd@outlook.com; 6 Department of Obstetrics and Gynecology, Valleywise Health, Phoenix, AZ 85008, USA; dean_coonrod@dmgaz.org; 7 Division of Maternal-Fetal Medicine, Oregon Health & Science University, Portland, OR 97239, USA; pereiral@ohsu.edu; 8 Department of Obstetrics & Gynecology, Christiana Care Health System, Newark, DE 19718, USA; mhoffman@christianacare.org

**Keywords:** gestational age, gestational age dating, preterm birth, spontaneous preterm birth, proteomic biomarker risk predictor

## Abstract

The clinical management of pregnancy and spontaneous preterm birth (sPTB) relies on estimates of gestational age (GA). Our objective was to evaluate the effect of GA dating uncertainty on the observed performance of a validated proteomic biomarker risk predictor, and then to test the generalizability of that effect in a broader range of GA at blood draw. In a secondary analysis of a prospective clinical trial (PAPR; NCT01371019), we compared two GA dating categories: both ultrasound and dating by last menstrual period (LMP) (all subjects) and excluding dating by LMP (excluding LMP). The risk predictor’s performance was observed at the validated risk predictor threshold both in weeks 19^1/7^–20^6/7^ and extended to weeks 18^0/7^–20^6/7^. Strict blinding and independent statistical analyses were employed. The validated biomarker risk predictor showed greater observed sensitivity of 88% at 75% specificity (increases of 17% and 1%) in more reliably dated (excluding-LMP) subjects, relative to all subjects. Excluding dating by LMP significantly improved the sensitivity in weeks 19^1/7^–20^6/7^. In the broader blood draw window, the previously validated risk predictor threshold significantly stratified higher and lower risk of sPTB, and the risk predictor again showed significantly greater observed sensitivity in excluding-LMP subjects. These findings have implications for testing the performance of models aimed at predicting PTB.

## 1. Introduction

Preterm birth (PTB), including both spontaneous (sPTB) and indicated delivery earlier than 37 weeks of gestational age (GA), is the leading global cause of perinatal morbidity and mortality [1]. Each year, PTB occurs in more than 10% of U.S. births [2,3]. For decades, these estimates have remained essentially unchanged, despite evolving medical technologies and clinical practices. The economic impact of PTB on the U.S. healthcare system is immense, estimated to exceed USD 25 billion annually [4]. Thus, effectively addressing PTB persists as a critical need.

PTB is an adverse outcome defined by a single endpoint: delivery before an established time period as measured by an estimate of GA [5]. Consequently, uncertainty in GA dating, defined as the variability observed between the estimated and actual GA, affects the observed performance of a predictor of PTB. Further, the clinical management of pregnancy relies on GA, which is set by establishing the estimated due date (EDD) following professional society recommendations and guidelines [6,7]. Conventionally, in the United States, the EDD is set at 280 days following the first day of the last menstrual period (LMP). However, LMP dating assumes a regular, 28-day menstrual cycle with ovulation on day 14 and set timing for implantation, though studies have shown that approximately half of all women do not recall their precise LMP date [7,8,9,10]. Even when the LMP is known, it is surprisingly uncertain in determining the EDD, with a 95% confidence interval of ±29 days [11,12]. Today, ultrasound measurements during the first trimester of pregnancy are considered the most certain method for establishing (or confirming) GA [7,8,9,13,14,15,16]. Ultrasound measurements through week 21 of pregnancy are regarded as standard in the obstetric estimation of EDD and can be used to confirm or replace an LMP-established EDD. Pregnancies dated by LMP without confirmation or revision based on ultrasound examination before week 22 of gestation are considered to show sub-optimal dating [7,17].

The successful application of any PTB-preventive strategy is enabled by the early and accurate identification of higher-risk pregnancies. Here, we consider the performance of a risk factor or predictor in terms of how well it identifies pregnancies destined for sPTB. A history of prior PTB and short cervical length in the current pregnancy are clinically accepted risk factors for sPTB but combine to detect less than 20% of singleton sPTBs [18,19]. A range of additional factors including body mass index (BMI), smoking, substance use and socioeconomic circumstances are commonly considered on a case-by-case basis in evaluating PTB risk but are not sufficiently prognostic for clinical use; instead, they are seen to provide opportunities for preconception and post-partum care. Untapped potential exists to develop tools, including molecular biomarkers, that sensitively identify PTB risk early in pregnancy, providing opportunities for risk-ameliorating interventions in addition to current options for acute care. Increasing true-positive and true-negative rates for prognostic tests improves the targeting of interventions and the allocation of resources, respectively.

Saade et al. [20] broadly validated a proteomic biomarker risk predictor for the assessment of sPTB risk in serum collected from asymptomatic singleton pregnancies in the United States at weeks 19^1/7^–20^6/7^ of gestation [21]. This risk predictor is based on the ratio of insulin-like-growth-factor-binding protein 4 (IBP4) and sex-hormone-binding globulin (SHBG). Clinical validation of the test was performed in an independent and representative set of women from the prospective Proteomic Assessment of Preterm Risk (PAPR) study (NCT01371019) [20], a large, multicenter, observational study that enrolled a diverse population across 14 U.S. sites, emphasizing academic medical centers. The PAPR analysis established a predictive biomarker threshold score that significantly stratifies premature from later GAs at birth and corresponds to a 15% risk, i.e., a twofold increase compared with the average risk across U.S. singleton pregnancies [6]. Subsequently, this threshold was validated in subjects from an independent, prospective cohort (Multicenter Assessment of a Spontaneous Preterm Birth Risk Predictor (TREETOP); NCT02787213) [22,23]. The prediction of health outcomes related to prematurity by these biomarkers also was confirmed in TREETOP [22].

The PAPR trial was concluded prior to the publication of current American College of Obstetricians and Gynecologists (ACOG) guidelines for GA dating [7]. The objectives of our current study were: (1) to estimate biomarker risk predictor performance more accurately by restricting the analysis of the PAPR cohort to women who have more certain GA dating as per current practice guidelines; and (2) to test the generalizability of the effect of dating certainty upon observed performance amongst these women, by comparing performance in the previously established blood draw window of 19^1/7^–20^6/7^ weeks’ GA with that for a broader GA window, 18^0/7^–20^6/7^ weeks.

## 2. Materials and Methods

The current study was a secondary analysis of the prospective PAPR clinical trial (NCT01371019), using only subjects held out for validation and not employed in the discovery or verification of biomarker prediction [20]. The PAPR study enrolled 5501 pregnant women between 17^0/7^ and 28^6/7^ weeks’ GA across 11 sites in the United States for the purpose of discovering and validating a biomarker prediction of spontaneous preterm delivery (sPTB). The PAPR study was approved by the Institutional Review Boards/Ethics Committees of all participating study sites. Informed consent was obtained from all subjects involved in the study. The PAPR study was conducted before the ACOG Committee Opinion 700 (CO 700), which provides guidance on GA dating, was issued [7].

In the current analysis, we compared the performance of the proteomic biomarker risk predictor as published for women dated using any available method [20] against test performance observed in the subset of women whose pregnancies were dated with more certainty. For the purposes of our current analysis, GA calculated directly from a first- or second- trimester ultrasound was considered more certain, while GA calculated using LMP was considered less certain, consistent with current practice standards [7]. To evaluate the generalizability of the effects of GA dating on observed test performance, we also compared risk predictor performance among more certainly dated subjects. These included subjects in both the previously established blood draw window of 19^1/7^–20^6/7^ weeks’ GA and in a broader GA window of 18^0/7^–20^6/7^ weeks, inclusive of subjects not previously assessed by these measures. The primary outcome measured was the predictive performance of the test, the endpoints for which included a regression test for sPTB case classifications, sensitivity, specificity, area under the receiver operating curve (AUC), positive predictive value (PPV) and negative predictive value (NPV), evaluated at the validated biomarker threshold score [20,23]. 

### 2.1. Study Population

The evaluated study population was the PAPR validation cohort [20], for which data were prospectively collected under a strict blinding protocol. The sample size was sufficient to power the study to >80%, assuming an AUC of 0.75 and an alpha of 0.05, and to power a regression test of classification at the validated threshold with 75% sensitivity and 74% specificity. BMI in the PAPR population was derived from height and prepregnancy self-reported weight and reported in two categories: (1) “All BMI”, representing the full range of BMI scores; and (2) “Stratified BMI”, which only included BMI scores in the range of >22–≤37 kg/m^2^. Deliveries were classified as term births (≥37^0/7^ weeks GA) or sPTBs. 

### 2.2. Gestational Age Dating and Estimated Delivery Date

The PAPR clinical trial protocol specified an algorithm for the assessment of GA and EDD. In recognition of the importance of dating certainty, the protocol specified that ultrasound was the preferred method of dating and, when possible, the earliest available ultrasound should be used for GA determination. LMP was to be used on its own only in the absence of other dating methods. When both ultrasound and LMP were available, subjects were dated using LMP if the LMP date was <7 days different from a 1st-trimester ultrasound date, <10 days different from an early 2nd-trimester ultrasound date (14^0/7^–20^0/7^), <14 days different from a late 2nd-trimester ultrasound date (20^1/7^–27^6/7^), or <21 days different from a 3rd-trimester ultrasound date. Among 4285 PAPR subjects who had a record of GA dating method, 37.3% were dated by a 1st-trimester ultrasound, 11.0% by an early 2nd-trimester ultrasound, 2.1% by a late 2nd-trimester ultrasound and 49.5% by LMP. We classified subjects with a record of direct use of LMP to establish the EDD as “LMP” and all others as “excluding LMP.” This was a conservative assumption, in that subjects without a record of a GA dating method were included in the excluding-LMP group. The population of subjects dated by any method (all subjects) was compared to the excluding-LMP subset population.

### 2.3. Sample Analysis

Samples were analyzed in a Clinical Laboratory Improvement Amendments, College of American Pathologists and New York State Department of Health certified laboratory according to a previously described standard operating protocol [20,21]. Briefly, serum samples were depleted of the 14 most abundant proteins, reduced, alkylated, and digested with trypsin. Samples then were spiked with stable isotope standard peptides for proteins of interest, desalted, and analyzed using reverse phase liquid chromatography, followed by multiple reaction monitoring mass spectrometry. Relative levels of IBP4 and SHBG were expressed as response ratios of the peak area for the endogenous peptide divided by the peak area of the stable isotopic standard peptide. The IBP4/SHBG proteomic biomarker was calculated as: ln(IBP4 response ratio/SHBG response ratio). Measurements within 10% of the standard analytic error (standard deviation of replicates) of the test were considered equivalent.

### 2.4. Statistical Analysis

All analyses of AUC, sensitivity, and specificity tested predefined hypotheses using a prespecified statistical analysis plan. The blinded assessment of hypotheses was conducted by a third-party statistician. In post hoc analyses, NPV and PPV were calculated from sensitivity, specificity, and an sPTB prevalence of 7.3%, as specified [20]. NPV and PPV confidence intervals were calculated as appropriate for a case–control study [24]. Means not contained within comparator 95% confidence intervals indicated significant differences in predictor performance metrics. Analyses were performed in R 3.5 or higher, using the packages data.table [25], pROC [26], and binom [27].

### 2.5. Estimation of the Effects of Certainty of Gestational Age Dating on Prediction of Prematurity

We simulated the effects of dating uncertainty on observed predictor performance using the 2019 distribution in the United States of GA at birth [28] and a simplification of intervals in guidelines for the use of ultrasound dates provided in ACOG CO 700 [7]. 

The United States’ national distribution of GA at birth for singleton pregnancies was retrieved from the CDC for 2019, the most recent full year of data not known to be affected by the COVID-19 pandemic [28]. Spline interpolation was used to convert CDC GAs at birth from weeks to days. 

ACOG guidelines’ intervals for the confirmation of LMP by ultrasound were used as the half-widths of 95% confidence intervals of ultrasound dates: 7 days for 1st-trimester dating and 10 days for 2nd-trimester dating. The two-standard-deviation interval for LMP dating has been reported to be 29 days for known LMP and 53 days for uncertain LMP [11]. Based on known similar centers [7,8] and independent spreads of LMP and ultrasound dating and the above standard deviations with the assumption of normally distributed values, we estimated that about half of LMP dates would be confirmed by a 2nd-trimester ultrasound, with a two-standard-deviation interval of 14 days for the confirmed LMP dates.

We defined a perfect predictor that assigned high risk probabilities to all births below 37 weeks of GA and low risk probabilities to all births at or above 37 weeks of GA. Random sets of 0.1% of births were selected 20 times. Each set was assigned GA dating types at prevalences observed in PAPR: half LMP confirmed by ultrasound, half pure ultrasound. Random normally distributed noise was added to the GAs at birth to simulate uncertainty in GA dating, calculated with a mean of zero and standard deviations derived from guidelines as established [7]. Lastly, the predictor perfectly matched to the original GAs was tested for the AUC of the prediction of PTB amongst the adjusted GAs. 

## 3. Results

Table 1 summarizes the characteristics of the subjects in the study, with comparisons between the all-subject population and the excluding-LMP population for the GA windows of weeks 18^0/7^–20^6/7^ and 19^1/7^–20^6/7^. No significant differences were observed between the two populations across a range of demographic and clinical parameters.

**Figure 1:**
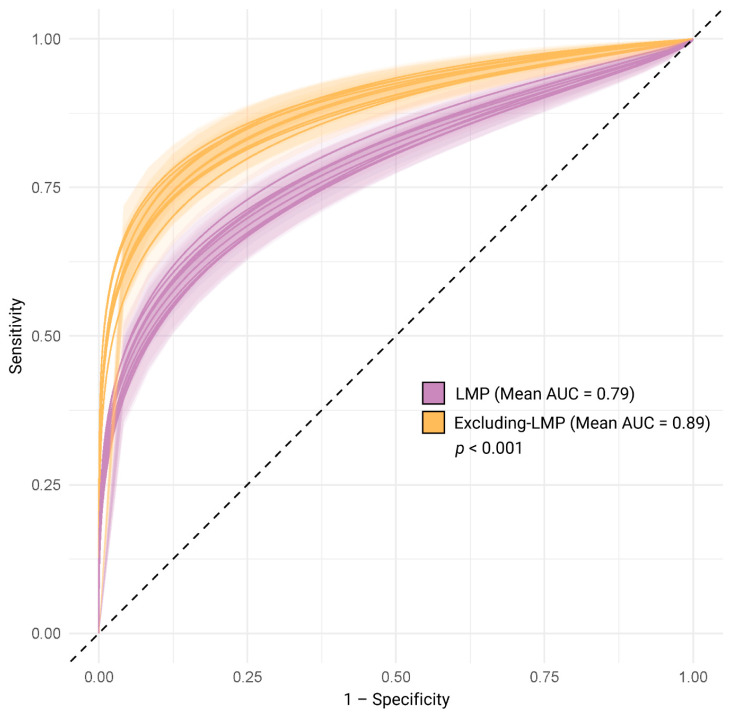
Performance of a hypothetical perfect preterm birth risk predictor using first date of last menstrual period (LMP) or excluding-LMP gestational age dating. Darker curves represent individual simulations, while the shaded area represents the 95% confidence interval of sensitivity at each value of 1 − specificity. AUC, area under the receiver operating curve.

Figure 1 shows the expected performance of a simulated perfect PTB predictor on subjects with GAs determined by LMP or excluding-LMP dating, interpreted as per ACOG CO 700 guidance. Performance was significantly lower with LMP than with excluding-LMP dating (mean LMP AUC: 0.79; mean excluding-LMP AUC: 0.89; *p*-value < 0.001).

Applying the ACOG estimates of reliability of dating to the present study, we estimated that in weeks 19^1/7^–20^6/7^, three births labeled as sPTB in the all-subject population and one in the excluding-LMP group were likely to have been term births, while less than one term birth in each was likely to be a misclassified PTB. In weeks 18^0/7^–20^6/7^, we estimated that at least one additional sPTB and one additional term birth were likely to have been misclassified.

### Risk Predictor Performance

The AUC of the proteomic biomarker sPTB risk predictor was significant in the validated draw window, weeks 19^1/7^–20^6/7^, for both all subjects (0.75) and excluding-LMP subjects (0.80) in the BMI-stratified population. Similarly, the correlation between the sPTB risk predictor and GA at birth was significant in both populations, with Pearson correlation coefficients −0.6 and −0.5 in the all-subject and excluding-LMP, BMI-stratified populations, respectively. At the validated threshold and the range of GA at blood draw reported in Saade et al. [28], the sPTB risk predictor showed previously reported performance within the all-subject BMI-stratified population, extended here with additional descriptive statistics: 75% sensitivity, 74% specificity, 18% PPV, and 97% NPV. At the same threshold in the excluding-LMP, BMI-stratified population, the sPTB risk predictor showed higher performance, with 88% sensitivity, 75% specificity, 22% PPV, and 99% NPV. The only significant difference in performance between the all-subject population and the excluding-LMP population was in sensitivity, although point estimates were generally numerically higher in the excluding-LMP population, while confidence intervals overlapped.

**Figure 2:**
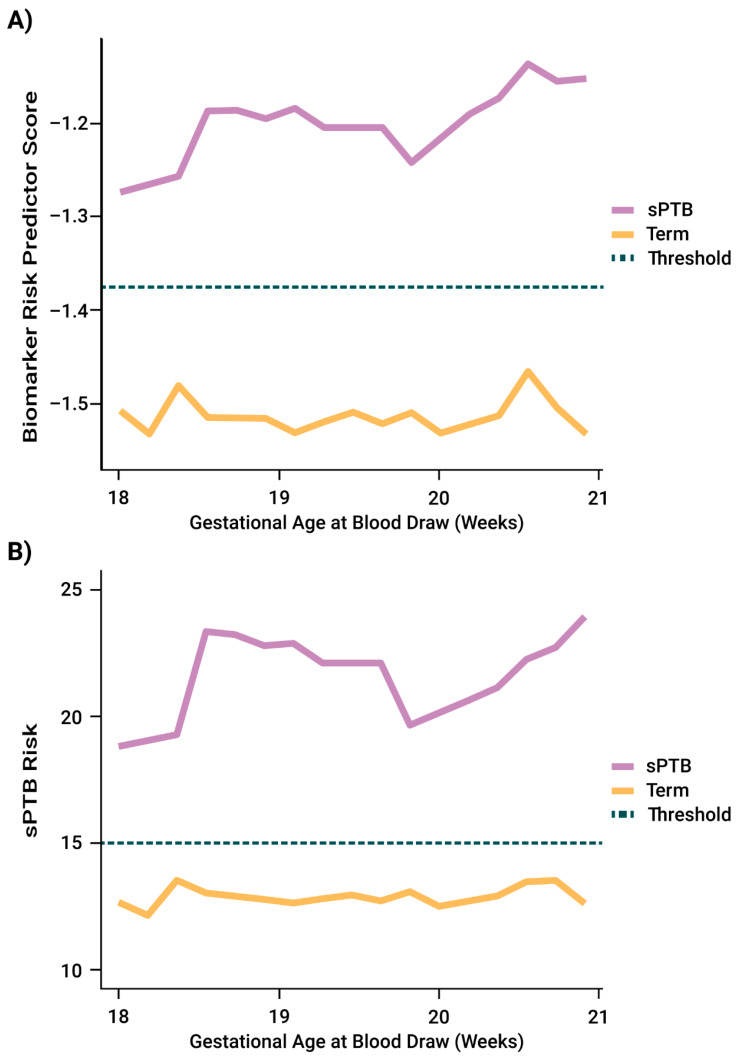
Separation between spontaneous preterm birth (sPTB) cases and term births (controls) across gestational age (GA) at blood draw, in the excluding-LMP (not dated by first day of last menstrual period) population. (**A**) Using the proteomic predictor score. Dashed line corresponds to the validated risk predictor threshold (−1.37), representing 15% sPTB risk, or twice the average sPTB risk across all U.S. singleton pregnancies. (**B**) Using the percent sPTB risk. Dashed line indicates 15% sPTB risk.

To test whether these observations extended to additional subjects whose samples were collected in a broader GA blood draw window, we compared the performance of the risk predictor in excluding-LMP subjects with blood drawn in weeks 19^1/7^–20^6/7^ versus that in weeks 18^0/7^–20^6/7^. As a baseline observation, we found that the validated threshold significantly stratified higher- from lower-risk subjects in weeks 18^0/7^–20^6/7^. Additionally, there was no significant difference in sPTB risk predictor performance in the excluding-LMP population in weeks 18^0/7^–20^6/7^ compared to 19^1/7^–20^6/7^. Sensitivity, specificity, PPV, and NPV at the validated threshold did not differ, nor did AUC and correlation to GA at birth. As well, values did not differ significantly by BMI stratification. However, sensitivity was significantly improved in weeks 18^0/7^–20^6/7^ in the excluding-LMP population as compared to the all-subject population. Specificity, NPV, PPV, AUC, and correlation to GA at birth showed numerical increases in point estimates in the excluding-LMP population relative to the all-subject population, with overlapping confidence intervals. Figure 2 shows the separation in risk predictor scores between sPTBs and term births (controls) for the excluding-LMP population across GA at blood draw, relative to (A) the proteomic biomarker risk predictor score and (B) the validated threshold.

## 4. Discussion

In the current analysis, we demonstrated an improvement in observed biomarker risk predictor performance in representative subjects who had more certain GA dating. The fact that subjects with more certain dating did not differ from all subjects by any demographic or clinical factor suggests that the improvement we observed in performance is only due to more certain dating and applies to all pregnancies, no matter how they are dated. We note that the sPTB risk predictor assessed in the current analysis was developed on a broad and diverse United States pregnant population and is applicable across demographic groups, including those based on race or ethnicity. Performance improvement also was confirmed in additional subjects by extending the analysis from the current intended-use window of 19^1/7^–20^6/7^ weeks to a broader window of 18^0/7^–20^6/7^ weeks.

Based on the lower reliability of their GA dating, we estimated that three term births in weeks 19^1/7^–20^6/7^ were misclassified as preterm when all dating methods were included, while only one was estimated to have been misclassified with more reliable dating. Thus, the significantly increased sensitivity that we observed at the validated risk predictor threshold is indeed the most likely result of restricting analysis to subjects with better dating. Our data suggest that lower-scoring cases contributing to the original, lower estimate of sensitivity largely had received less certain dating and that at least some are expected to represent term births misclassified as PTBs due to dating uncertainty.

ACOG guidance regarding GA dating was revised after PAPR study data were collected, providing new specifications for the uncertainty of available GA dating methods [7]. These specifications motivated the current analysis. ACOG guidance quantifies the increased certainty of GA dating with earlier GA at ultrasound. More certain dating in turn provides greater certainty for GA-related outcomes such as PTB and thus provides more accurate quantitation of risk predictor performance. 

The impact of GA dating uncertainty on the assessment of the prediction of GA-dependent events such as PTB can be quantified. In our simulations, a perfect PTB predictor showed a decrease in the AUC of 21% when GA was determined by LMP dating confirmed by ultrasound and about half that decrease when GA was determined by ultrasound dating. This simulation demonstrates the inaccuracy of assessing predictor performance in a population for which the outcome (sPTB as determined by GA date) is not known reliably. While ultrasound dating is commonly accepted as a more certain dating method than LMP, our results demonstrate the novel suggestion that confirming LMP by ultrasound does not improve its certainty to the level achieved by using actual ultrasound dates. The impact of GA dating certainty can also be quantified in ways that impact daily obstetric practice. Based on the approximately nine-fold higher prevalence of term than PTBs, less certain GA dating notably increases the number of term births misclassified as preterm, while a smaller number of PTBs will be misclassified as term births. Estimated GAs that provide higher numbers of false positive and false negative calls for PTB result in more opportunities for the incorrect application of treatments such as antenatal corticosteroid administration. 

Uncertainty in GA dating may be particularly impactful upon medical decisions for preterm and late-term or post-date deliveries. Maternal and neonatal care recommendations may differ strongly with threatened labor or delivery at an estimated GA of 21^6/7^ vs. 22^6/7^ weeks. Similarly, recommendations for intervention as opposed to expectant management may differ for post-term pregnancy at 41^0/7^ vs. 42^0/7^ weeks. Such challenging scenarios motivate the development of prognostics or diagnostics that can improve the certainty of GA dating beyond the current state and thus improve the performance of GA as a classifier of risk of periviable or post-term birth. These findings have wider-ranging implications beyond PTB prediction and may affect the timing of antenatal testing and induction, reductions in cesarean section, and the prevention of stillbirth. The results of the present analysis suggest that use of pure ultrasound dating with a validated proteomic biomarker risk predictor may allow the most accurate assessment of the prediction of PTB. As well, combinations of biomarkers selected for the estimation of GA at the time of sampling rather than risk prediction, in combination with ultrasound, may be of interest for the more confident estimation of GA and EDD. 

Future work might include an examination of the observed performance of pregnancy predictors on additional cohorts with two or more GA dating techniques assessed on all subjects, enabling within-subject comparison of the effects of dating uncertainty on performance assessment.

The limitations of the current study include the modest size of the study and the availability of only one GA dating method per subject. In addition, the exact GA of the dating ultrasound was not available in the PAPR study. For this reason, we established biomarker performance amongst a more precisely dated population by excluding LMP-only dating. Future studies are planned to extend these analyses in clinical trials where the gestational age of the dating ultrasound is available and within-patient comparison of gestational age dating methods can be carried out. Finally, ours was a retrospective analysis, which can be enhanced by focused prospective studies.

A major strength of the study was that it applied the current best practices, including the implementation of ACOG guidance and evidence cited therein and a blinded analysis by a third-party statistician. The analysis was conducted on a well-characterized, previously studied population. Finally, the current study introduced a methodology for assessing risk predictor performance more accurately through the consideration of GA dating uncertainty.

## 5. Conclusions

The improved estimation of the performance of an sPTB risk predictor in subjects whose GA at delivery is more certain suggests that the risk predictor provides accurate predictions that are confirmed by better dating. Improvements in risk prediction can lead to better risk stratification, and this work suggests that more well-designed controlled studies on interventions to reduce risk are warranted and have the potential to have significant impacts.

## Figures and Tables

**Table 1:** Demographic comparison of all-subject and excluding-LMP (not dated by first day of last menstrual period) populations in gestational age weeks 19^1/7^–20^6/7^ and 18^0/7^–20^6/7^.

		Weeks 19^1/7^–20^6/7^			Weeks 18^0/7^–20^6/7^		
Demographic/ Clinical Variable	Value	All- Subjects	Excluding LMP	*p*-Value	All Subjects	Excluding LMP	*p*-Value
**Maternal age**	Median (IQR)	24.5 (21.0–30.0)	23 (21.0–28.0)	0.72	24.5 (22.0–31.0)	23.0 (21.5–28.0)	0.7
**Maternal BMI**	Median (IQR)	26.5 (22.3–31.3)	28.5 (23.8–34.6)	0.7	28.0 (23.5–32.0)	29.4 (24.4–34.6)	0.7
**Gravida**	Primigravida	13	7	1	24	11	0.83
	Multigravida	41	22		60	33	
**Race**	Black	13	5	0.76	17	8	0.95
	White	38	23		61	33	
	Other	3	1		6	3	
**Ethnicity**	Hispanic	22	12	0.97	36	20	0.9
	Non-Hispanic	32	17		48	24	
**Prior PTB**	No	34	19	0.9	50	29	0.76
	Yes	20	10		34	15	
**GABD**	Median (IQR)	139 (135–144)	139 (135–144)	0.96	135 (130.5–142.5)	135 (130–143)	0.98
**GAB**	Median (IQR)	273 (256–281)	273 (258–277)	0.98	273 (256–281)	273 (257–277)	0.96
**Neonatal gender**	Female	22	21	0.34	39	17	0.46
	Male	32	8		45	27	
**Outcome**	Cases	18	10	0.95	28	15	0.97
	Controls	36	19		56	29	
	Total	54	29		84	44	

Continuous data: 2-sided Wilcoxon test. Medians and IQRs are shown. Categorical data: 2-sided Fisher’s Exact test. Counts are shown. IQR, interquartile ratio; excluding LMP, not dated by first day of last menstrual period. GAB, gestational age at birth; GABD, gestational age at blood draw; LMP, last menstrual period; PTB, preterm birth.

## Data Availability

Data supporting the results presented here are available on request from the corresponding author. Data will not be made publicly available or in any format that may violate a subject’s right to privacy. For example, dating information or identifiers that could allow data integration, thereby enabling potential identification of study subjects, are protected.
